# Household air pollution and urinary incontinence symptoms among older adults in LASI: a large-scale population-based study

**DOI:** 10.1186/s12889-024-18834-x

**Published:** 2024-05-31

**Authors:** Xiaoyang Liu, Kai Ma, Shengzhuo Liu, Pan Song, Luchen Yang, Zhenghuan Liu, Jing Zhou, Linchun Wang, Xin Yan, Yunfei Yu, Qiang Dong

**Affiliations:** Department of Urology, Institute of Urology, West China Hospital, Sichuan University, Chengdu, China

**Keywords:** Urinary incontinence, Solid fuel use, Indoor pollution, India

## Abstract

**Background:**

The effects of household air pollution on urinary incontinence (UI) symptoms and stress urinary incontinence (SUI) symptoms have not been studied. This study seeks to investigate the correlation between household air pollution and UI/SUI symptoms among middle-aged and elderly adults in India.

**Methods:**

We employed data derived from individuals aged 45 years and older who participated in the inaugural wave (2017–2018) of the Longitudinal Aging Study in India (LASI). The assessment of household air pollution exposure and the occurrence of UI/SUI symptoms relied on self-reported data. The analytical approach adopted was cross-sectional in nature and encompassed a cohort of 64,398 participants. To explore relationships, we utilized multivariate logistic regression analysis, incorporating subgroup analysis and interaction tests.

**Results:**

1,671 (2.59%) participants reported UI symptoms and 4,862 (7.55%) participants reported SUI symptoms. Also, the prevalence of UI/SUI symptoms is much higher among middle-aged and elderly adults who use solid polluting fuels (UI: 51.23% vs. 48.77%; SUI: 54.50% vs. 45.50%). The results revealed a noteworthy correlation between household air pollution and the probability of experiencing UI/SUI symptoms, persisting even after adjusting for all conceivable confounding variables (UI: OR = 1.552, 95% CI: 1.377–1.749, *p* < 0.00001; SUI: OR: 1.459, 95% CI: 1.357–1.568, *p* < 0.00001). Moreover, significant interaction effects were discerned for age, education level, tobacco consumption, alcohol consumption, and physical activity (p for interaction < 0.05).

**Conclusions:**

The results of our study indicate that the utilization of solid fuels in the home increases the likelihood of developing urinary incontinence and stress urinary incontinence. As a result, we argue that there is an immediate need to reform the composition of cooking fuel and raise public awareness about the adverse effects of air pollution in the home.

## Introduction

 It has been reaffirmed by the United Nations Climate Change Conference that immediate global action is required to mitigate climate change [1]. Air pollution has long been known to pose a serious risk to several aspects of human health, such as respiratory and allergy disorders, particularly in low- and middle-income countries (LMICs) [1]. Air pollution significantly contributes to climate change by means of the escalating concentrations of greenhouse gases. In addition to its negative effects on the ecosystem, exposure to air pollution may have long-term health impacts such as lung cancer, heart disease, and respiratory diseases [2]. According to GBD studies, exposure to ambient and household PM2.5 is one of the biggest single causes of premature mortality in India and recent studies have estimated that the primary source of ambient PM2.5 exposure in the nation is household emissions [3, 4]. Nevertheless, the majority of research has been devoted to examining the negative consequences of ambient air pollution, with relatively few studies delving into the detrimental effects of domestic air pollution. According to WHO, around 2.6 billion individuals globally depend on solid fuels for their cooking needs, leading to an estimated 3.8 million early deaths [2]. In light of its substantial demographic size and the pronounced challenge of an aging population, India is experiencing a noteworthy surge in its demographic numbers and 57% of rural Indian households still rely on solid fuels for cooking [5, 6], pp. 2001–2051).

Urinary incontinence is a commonly seen urologic disorder in the elderly population, characterized by the involuntary release of urine. This condition has significant implications for the physical, psychological, and social well-being of affected individuals, leading to functional impairments and a reduced overall quality of life [7–9]. At the same time, as one of the most common conditions of urinary incontinence (UI), stress Urinary Incontinence (SUI) is the inability to control the leakage of urine during increased abdominal pressure, such as coughing, sneezing or lifting weights. The bladder serves as a reservoir, while the bladder outlet functions as a sphincteric mechanism, making up the lower urinary tract. It is also a complicated sequence of precisely regulated and integrated neuromuscular actions involving anatomic and neurologic systems. UI/SUI may be caused by changes to any of these elements, and its frequency rises with aging [9].

To the best of our knowledge, the potential effects of household air pollution on UI/ SUI symptoms remain unexplored in the existing literature. The principal objective of this study was to examine the influence of indoor air pollution on the manifestation of UI and SUI symptoms among older and middle-aged adults in India. To improve the practicality of our findings, our study employed nationally representative data derived from Longitudinal Aging Study in India (LASI) Wave 1. Moreover, this study not only incorporate doctors’ diagnosis as the criteria for UI, we also added self-reported symptoms of stress incontinence to further affirm our results. As a result, we found that indoor air pollution may increases the risk of getting UI/SUI symptoms after adjusting for all variables (UI: OR: 1.552; 95% CI: 1.377–1.749, *P* < 0.001; SUI: OR: 1.459; 95% CI: 1.357–1.568, *P* < 0.001). We also proposed that this association may be a result of oxidative stress (OS) and metabolic disturbance [10–12]. Systemic inflammation and oxygen free radicals can cause oxidative damage to bladder cells’ membranes and urethra nerve cells. Also, the interaction between fuel particulate matter and chemical substances has been associated with metabolic disorders, influencing hormonal secretion, autonomic nervous system equilibrium, and vasopressin secretion, which play a pivotal role in modulating the regulation of smooth muscle tone [8, 11, 13].

The principal objective of our study was to investigate potential correlations between indoor air pollution and the prevalence of UI/SUI symptoms. Additionally, the study sought to raise awareness among policymakers on the necessity to endorse sustainable and economically viable alternatives to indoor air pollution sources. Lowering indoor air pollution can improve quality of life as people age by reducing health effects, particularly for the vulnerable older adult population.

## Methods

### Data resource

The data utilized in this study were obtained from LASI Wave 1, a comprehensive nationwide survey examining the economic, physiological, social and psychological well-being of senior individuals in India. LASI Wave 1 is aligned with the global Health and Retirement Study (https://g2aging.org/). A total of 73,396 individuals aged 45 years and older, providing a comprehensive representation of India and its 35 states or union territories, was included in LASI Wave1. It adopted a meticulous multistage stratified area probability cluster sampling design for the selection of participants.

**Fig. 1:**
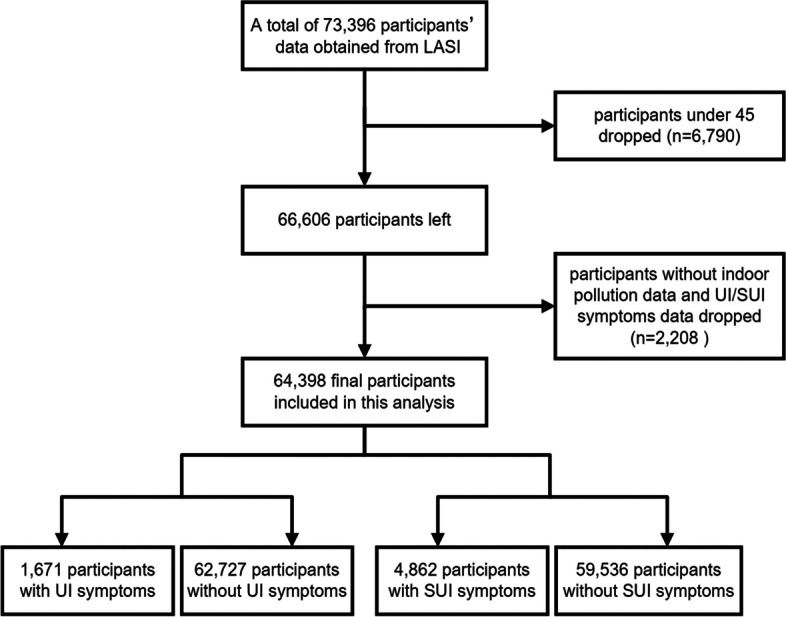
Flowchart of the participants selection

 A three-stage sampling design was implemented for rural areas, whereas a four-stage sampling design was employed for urban areas in each state. Each participant expressed formal assent by affixing their signature to a documented consent form (detailed contents available at official website). Figure 1 illustrates the detailed enrollment process of our study.

### Definition of household air pollution and urinary incontinence symptoms

In the LASI research, participants were instructed to provide information on their primary source of cooking fuel by responding to the question:” What is your main source of cooking fuel?” Individuals who self-reported the utilization of clean fuels, including liquefied petroleum gas, biogas, or electricity, were classified as clean fuel users. Conversely, participants who indicated the usage of alternative fuels were categorized as solid fuel users. Symptoms of urinary incontinence and stress urinary incontinence (UI/SUI) were self-reported and evaluated through the presentation of the inquiry: “Have you ever been diagnosed with any of the following urogenital conditions or diseases with options including urinary incontinence?” and “Do you ever pass urine while sneezing, coughing, laughing or lifting heavy objects?”

### Covariates

This study integrated specific parameters that have been shown to affect the onset of UI/SUI symptoms to mitigate the influence of potential confounding variables. The patients were categorized into four age groups: <60, < 70, <80, and ≥ 80 years old. The educational levels were categorized into four distinct classifications: above higher secondary, secondary and higher secondary, middle school, or under and never educated. Given India’s distinctive ethnic composition, the participants were stratified into 4 categories. Marital status was delineated into three categories: and residence were classified into rural and urban. In addition, Body Mass Index (BMI) categories were defined as follows: underweight (< 18.5 kg/m²), normal (18.5–25 kg/m²), and overweight (> 25 kg/m²). Classifications were established according to the frequency of alcohol consumption, physical activity levels and cigarette consumption (heavy, moderate, light, never). Additionally, a multitude of chronic disorders has been identified as potential confounding factors through the inquiry: “Has a health professional ever diagnosed you with any of the following chronic conditions or diseases?“. The study encompassed antecedent medical diagnoses, including but not limited to cardiovascular diseases, respiratory illnesses, hypertension, hyperlipidemia, malignancy and neurological conditions. To address the absence of certain covariates, R package mice (Multiple Imputation by Chained Equations approach), a widely recognized and effective method for handling missing data in epidemiological studies [14], was employed in our study. This technique allows us to impute missing values for multiple variables simultaneously, preserving the relationships between variables and improving the accuracy of estimates.

### Statistical analysis

Standard deviations and means are utilized as measures of central tendency and dispersion for continuous variables that adhere to a normal distribution in the baseline characteristics, while the interquartile range and median are employed to represent continuous variables that conform to a skewed distribution. Rates and percentages are used to depict categorical variables. The Kruskal-Wallis (K-W) test was employed to calculate *p*-values for continuous variables conforming to a skewed distribution. Conversely, chi-square tests were deployed for the analysis of categorical data. The Fisher exact test was employed in instances where the theoretical number was less than 10 [15].

In the current study, participants were stratified into two distinct groups based on their utilization of cooking fuel. The association between household air pollution and symptoms of urinary incontinence was examined by using multivariate logistic regression analysis. And multiple covariates were incorporated to mitigate the potential influence of confounding factors on the study’s outcomes. The Model I was exclusively adjusted for age groups. In contrast, Model II underwent adjustments for a more comprehensive set of covariates, namely age groups, caste/tribe, residence, marital status, and education level. Furthermore, Model III incorporated additional variables, namely tobacco consumption, alcohol consumption, and physical activity, into the previously adjusted covariates of Model II. Model IV was refined to include an extended array of antecedent medical diagnoses mentioned before. Interaction studies were performed in order to investigate the variability in the relationship between home air pollution and symptoms of incontinence, with the data stratified by variables. Subgroup analyses were performed utilizing stratified logistic regression models. The p-value for interaction was computed through the log-likelihood ratio test., necessitating a comparison between models that incorporated covariate interactions and those that did not. In the present study, a significance level of 0.05 was deemed as the threshold for statistical significance in relation to all reported statistical outcomes. The analyses were conducted using R version 4.2.2.

## Results

### Baseline characteristics

The study included a total of 64,398 participants, as outlined in Table 1. Among them, 1,671 participants reported symptoms of urinary incontinence (UI), and 4,862 participants reported symptoms of stress urinary incontinence (SUI). Notably, at baseline, participants with UI/SUI symptoms exhibited a higher prevalence of solid fuel usage compared to those without UI/SUI symptoms (UI: 51.23% vs. 45.75%; SUI: 54.50% vs. 45.19%). Concurrently, an observable trend indicated that participants with UI/SUI symptoms were of significantly older age compared to their counterparts without UI/SUI symptoms (UI: 64.56 ± 11.68 vs. 59.45 ± 10.48; SUI: 63.84 ± 11.51 vs. 59.24 ± 10.39). Additionally, significant differences (*P* < 0.001) were observed in diverse demographic and health-related variables. including caste/tribe, marital status, educational levels, alcohol consumption, physical activities, hypertension, diabetes, tumor, chronic lung disease, chronic heart disease, high cholesterol, stroke, bone or joint disease, and neurological or psychiatric problems between the groups with UI/SUI symptoms and those without.

**Table 1:** Baseline characteristics of the participants selection (IQR: interquartile range; for continuous variables: *P* value was calculated by Kruskal Wallis rank-sum test, Number (%) for categorical variables: *P* value was calculated by chi-square test)

		Urinary incontinence symptoms		*P*	Stress urinary incontinence symptoms		*P*
		No	Yes		No	Yes	
		*n* = 62,727	*n* = 1671		*n* = 59,536	*n* = 4862	
Gender (%)	Male	29,278 (46.68)	739 (44.23)	0.05	28,509 (47.89)	1508 (31.02)	< 0.001
	Female	33,449 (53.32)	932 (55.77)		31,027 (52.11)	3354 (68.98)	
Age (median [IQR])		58.00 [50.00, 66.00]	65.00 [55.00, 73.00]	< 0.001	58.00 [50.00, 66.00]	63.00 [55.00, 72.00]	< 0.001
Age class (%)	< 60	33,275 (53.05)	569 (34.05)	< 0.001	32,070 (53.87)	1774 (36.49)	< 0.001
	< 70	18,104 (28.86)	521 (31.18)		17,117 (28.75)	1508 (31.02)	
	< 80	8418 (13.42)	378 (22.62)		7736 (12.99)	1060 (21.80)	
	>=80	2930 (4.67)	203 (12.15)		2613 (4.39)	520 (10.70)	
Cooking fuel (%)	Clean	34,029 (54.25)	815 (48.77)	< 0.001	32,632 (54.81)	2212 (45.50)	< 0.001
	Solid	28,698 (45.75)	856 (51.23)		26,904 (45.19)	2650 (54.50)	
Caste tribe (%)	Scheduled caste	10,432 (16.77)	278 (16.79)	< 0.001	9899 (16.77)	811 (16.78)	< 0.001
	Scheduled tribe	11,106 (17.86)	188 (11.35)		10,238 (17.35)	1056 (21.85)	
	Other backward class	23,856 (38.35)	595 (35.93)		22,855 (38.72)	1596 (33.02)	
	No or other caste	16,805 (27.02)	595 (35.93)		16,030 (27.16)	1370 (28.35)	
Residence (%)	Rural	40,929 (65.25)	1064 (63.67)	0.191	38,696 (65.00)	3297 (67.81)	< 0.001
	Urban	21,798 (34.75)	607 (36.33)		20,840 (35.00)	1565 (32.19)	
Marital status (%)	Married	47,014 (74.95)	1138 (68.10)	< 0.001	45,058 (75.68)	3094 (63.64)	< 0.001
	Widowed	13,624 (21.72)	484 (28.96)		12,475 (20.95)	1633 (33.59)	
	Others	2087 (3.33)	49 (2.93)		2001 (3.36)	135 (2.78)	
Education level (%)	Never	29,469 (46.98)	769 (46.02)	< 0.001	27,499 (46.19)	2739 (56.33)	< 0.001
	Middle school or under	21,359 (34.05)	655 (39.20)		20,452 (34.35)	1562 (32.13)	
	Secondary and higher secondary	8623 (13.75)	192 (11.49)		8390 (14.09)	425 (8.74)	
	Above higher secondary	3275 (5.22)	55 (3.29)		3194 (5.36)	136 (2.80)	
BMI (%)	Underweight	10,521 (18.35)	308 (19.94)	0.212	9858 (18.12)	971 (21.72)	< 0.001
	Normal	30,043 (52.40)	807 (52.23)		28,654 (52.66)	2196 (49.13)	
	Overweight	16,770 (29.25)	430 (27.83)		15,897 (29.22)	1303 (29.15)	
Tobacco consumption (%)	Never	39,784 (63.76)	914 (55.03)	< 0.001	37,743 (63.74)	2955 (61.07)	< 0.001
	Light	9243 (14.81)	277 (16.68)		8851 (14.95)	669 (13.83)	
	Moderate	11,279 (18.08)	387 (23.30)		10,621 (17.94)	1045 (21.60)	
	Vigorous	2088 (3.35)	83 (5.00)		2001 (3.38)	170 (3.51)	
Alcohol consumption (%)	Never	51,169 (82.00)	1420 (85.49)	< 0.001	48,418 (81.75)	4171 (86.20)	< 0.001
	Light	6427 (10.30)	176 (10.60)		6135 (10.36)	468 (9.67)	
	Moderate	3231 (5.18)	48 (2.89)		3133 (5.29)	146 (3.02)	
	Heavy	1578 (2.53)	17 (1.02)		1541 (2.60)	54 (1.12)	
Physical activity (%)	Never	37,496 (60.09)	1152 (69.36)	< 0.001	35,398 (59.78)	3250 (67.18)	< 0.001
	Seldom	5438 (8.72)	136 (8.19)		5148 (8.69)	426 (8.81)	
	Sometimes	4381 (7.02)	105 (6.32)		4193 (7.08)	293 (6.06)	
	Frequent	15,080 (24.17)	268 (16.13)		14,479 (24.45)	869 (17.96)	
Hypertension (%)	No	44,852 (71.52)	948 (56.77)	< 0.001	42,772 (71.85)	3028 (62.32)	< 0.001
	Yes	17,864 (28.48)	722 (43.23)		16,755 (28.15)	1831 (37.68)	
Diabetes (%)	No	54,870 (87.50)	1282 (76.72)	< 0.001	52,058 (87.46)	4094 (84.26)	< 0.001
	Yes	7842 (12.50)	389 (23.28)		7466 (12.54)	765 (15.74)	
High cholesterol (%)	No	60,610 (96.63)	1565 (93.66)	< 0.001	57,541 (96.66)	4634 (95.33)	< 0.001
	Yes	2111 (3.37)	106 (6.34)		1990 (3.34)	227 (4.67)	
Tumor (%)	No	62,325 (99.37)	1649 (98.68)	0.001	59,168 (99.39)	4806 (98.89)	< 0.001
	Yes	396 (0.63)	22 (1.32)		364 (0.61)	54 (1.11)	
Chronic lung disease (%)	No	59,350 (94.62)	1459 (87.31)	< 0.001	56,423 (94.78)	4386 (90.23)	< 0.001
	Yes	3372 (5.38)	212 (12.69)		3109 (5.22)	475 (9.77)	
Chronic heart diseases (%)	No	60,527 (96.50)	1550 (92.76)	< 0.001	57,515 (96.61)	4562 (93.85)	< 0.001
	Yes	2195 (3.50)	121 (7.24)		2017 (3.39)	299 (6.15)	
Stroke (%)	No	61,733 (98.42)	1591 (95.21)	< 0.001	58,608 (98.45)	4716 (97.02)	< 0.001
	Yes	988 (1.58)	80 (4.79)		923 (1.55)	145 (2.98)	
Bone or joint diseases (%)	No	53,841 (85.84)	1206 (72.17)	< 0.001	51,183 (85.97)	3864 (79.49)	< 0.001
	Yes	8882 (14.16)	465 (27.83)		8350 (14.03)	997 (20.51)	
Neurological or psychiatric problem (%)	No	61,448 (97.98)	1581 (94.61)	< 0.001	58,371 (98.06)	4658 (95.84)	< 0.001
	Yes	1265 (2.02)	90 (5.39)		1153 (1.94)	202 (4.16)	

### Association between household air pollution and UI/SUI symptoms

Table 2 displays the outcomes of multivariate logistic regression analyses aimed at exploring the correlation between household air pollution and the probability of experiencing symptoms associated with UI and SUI symptoms among the study participants. In the crude model, the analysis revealed that older individuals utilizing solid fuels exhibited an elevated risk of experiencing UI/SUI symptoms (UI: OR: 1.245; 95% CI: 1.13–1.373, *P* < 0.001; SUI: OR: 1.453; 95% CI: 1.37–1.541, *P* < 0.001). After controlling for age and gender, the OR in Model I demonstrated statistical significance with a value of 1.557 (UI: OR: 1.217; 95% CI: 1.104–1.342, *P* < 0.001; SUI: OR: 1.43; 95% CI: 1.348–1.518, *P* < 0.001). The associations observed between the variables in Models II (UI: OR = 1.518; 95% CI: 1.352–1.705, *P* < 0.001; SUI: OR = 1.42; 95% CI: 1.342–1.524, *P* < 0.001) and III (UI: OR = 1.44; 95% CI: 1.281–1.62, *P* < 0.001; SUI: OR = 1.368; 95% CI: 1.274–1.468, *P* < 0.001) remained consistently robust, demonstrating resilience even after adjusting for various potential confounding factors. Following the adjustment for all potential variables in Model IV, the results maintained statistical significance (UI: OR: 1.552; 95% CI: 1.377–1.749, *P* < 0.001; SUI: OR: 1.459; 95% CI: 1.357–1.568, *P* < 0.001). Based on the findings of Model I, it can be extrapolated that age may exert a potential influence on the association between household air pollution and the likelihood of developing symptoms related to urinary incontinence (UI) or stress urinary incontinence (SUI). Model III posits that the observed association may be collectively influenced by alcohol consumption, cigarette consumption and physical activity. The results obtained from Model IV suggest that the presence of comorbidities may impact the overall significance of these associations.

**Table 2:** Multivariate logistic regression model of the relationship between household air pollution and UI/SUI symptoms (OR odd ratio, CI confidence interval, UI urinary incontinence, SUI stress urinary incontinence)

Exposure	Crude model		Model I		Model II		Model III		Model IV	
	OR (95%CI)	*P*-value	OR (95%CI)	*P*-value	OR (95%CI)	*P*-value	OR (95%CI)	*P*-value	OR (95%CI)	*P*-value
UI	1.245(1.13,1.373)	*p* < 0.0001	1.217(1.104,1.342)	*p* < 0.0001	1.518(1.352,1.705)	*p* < 0.0001	1.440(1.281,1.620)	*p* < 0.0001	1.552(1.377,1.749)	*p* < 0.0001
SUI	1.453(1.37,1.541)	*p* < 0.0001	1.430(1.348,1.518)	*p* < 0.0001	1.420(1.324,1.524)	*p* < 0.0001	1.368(1.274,1.468)	*p* < 0.0001	1.459(1.357,1.568)	*p* < 0.0001

Crude model adjust for none;

Model I adjust for: age; gender

Model II adjust for: age; gender; caste; residence; marital status; education level;

Model III adjust for: age; gender; caste; residence; marital status; education level; tobacco consumption; alcohol consumption; physical activity

Model IV adjust for: age; gender; caste; residence; marital status; education level; tobacco consumption; alcohol consumption; physical activity; BMI; Hypertension; Diabetes; High cholesterol; Tumor; Chronic lung disease; Chronic heart diseases; Stroke; Bone or joint diseases; Neurological or psychiatric problem

### Subgroup analysis

**Fig. 2:**
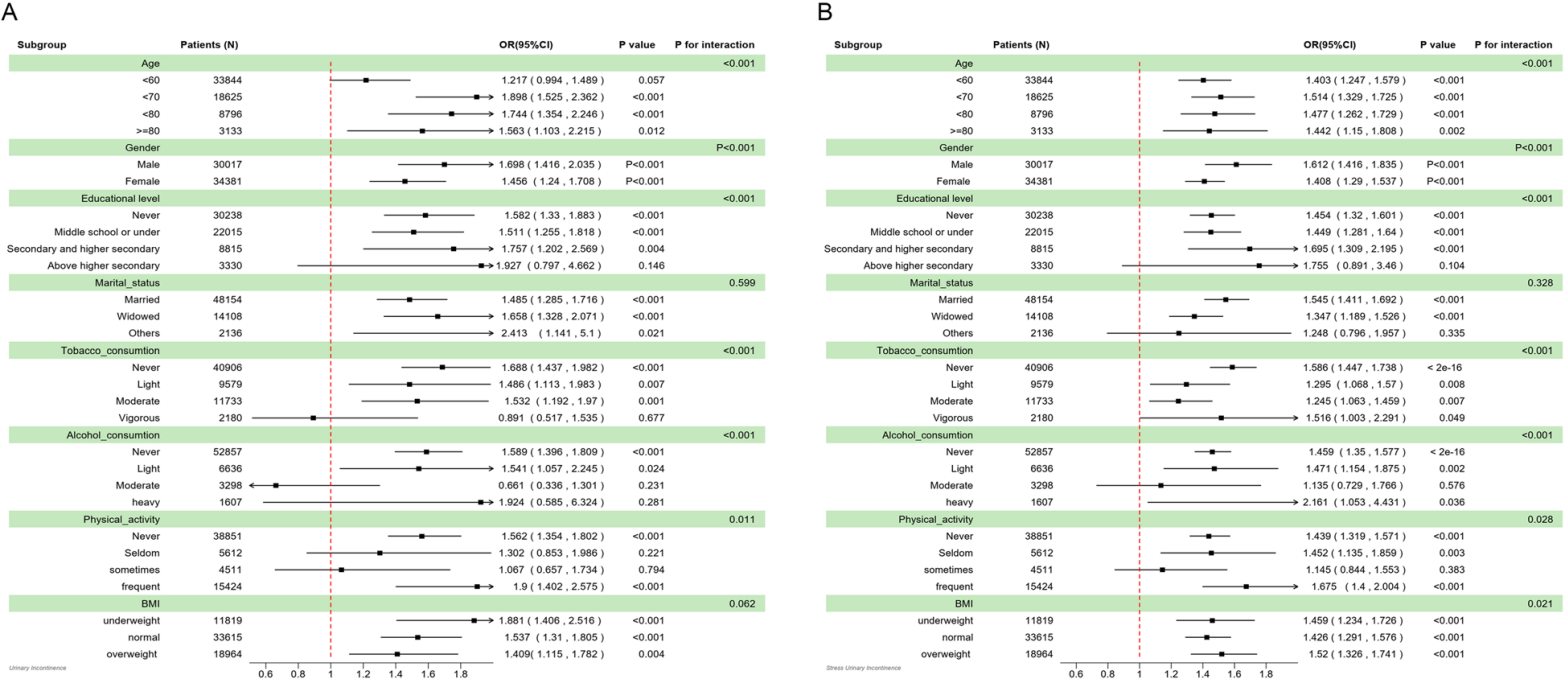
Subgroup analysis between household air pollution and the UI/SUI symptoms, **A **urinary incontinence **B **stress urinary incontinence; OR: odds ratio; 95% CI: 95% Confidence interval; BMI: body mass index; underweight: BMI < 18.5 kg/m2; normal: 18.5 kg/m2 ≤ BMI < 25 kg/m2; overweight: BMI ≥ 25 kg/m2

The results presented in Fig. 2 reveal a noteworthy correlation between household air pollution and the probability of experiencing UI and SUI symptoms among all age segments except for UI in < 60 subgroup. As for gender, it remains a noteworthy correlation in both male and female subgroups. Furthermore, as for education level subgroups, this correlation remained consistent except for above higher secondary subgroup. As for UI symptoms, this correlation remained consistent across individuals with varying levels of tobacco consumption (never, light, and moderate), alcohol consumption (never and light), and engagement in never and frequent physical activities. As for SUI symptoms, this relationship held consistent across gender; all the tobacco consumption; alcohol consumption (never, light and heavy); physical activity (never, seldom, and frequent); and all the BMI subgroups. The outcomes of the interaction test reveal a statistically significant influence of age, tobacco consumption, alcohol consumption, education level and physical activity on the association between household air pollution and symptoms of UI and SUI, which substantiated by a statistically significant p-value for interaction.

## Discussion

The global trend towards population aging is currently intensifying, marked by a discernible increase in the proportion of older individuals [16]. India, characterized by a vast population and a pronounced aging demographic, is currently undergoing substantial population growth. This growth is concomitant with a notable upswing in the proportion of individuals aged 60 and above, a trend anticipated to transpire from 2001 to 2031. The anticipated demographic shift is projected to manifest in an approximately twofold increase, reaching approximately 20% by the mid-century [17]. LASI, initiated in 2017–2018 as India’s inaugural research endeavor on aging, aims to comprehensively examine the economic, social, physiological, and psychological dimensions of the aging phenomenon within the country. This study affords us the opportunity to elucidate the intricate interplay between these multifaceted factors. Air pollution has been widely recognized as a significant threat to public health, particularly in low- and middle-income countries [18]. Previous research has offered insights into the associations between air pollution and chronic illnesses. Additionally, it has revealed that air pollution elevates the probability of adverse health conditions [10, 19], p. 1; Liu et al [20].

Previous studies have investigated the adverse effects of urinary incontinence symptoms on daily life, primarily focusing on general health status and mental well-being [21, 22]. A correlation between environmental pollutants and symptoms of urinary incontinence has been substantiated by several preceding studies. In Ni’s study, 4,406 female volunteers were evaluated for their exposure to cadmium, lead, and mercury (nephrotoxic metals) and their incidence of stress urinary incontinence and urgency urinary incontinence [23]. They discovered that cadmium was linked to an increased risk of UI and that the incidence of SUI rose as cadmium levels in blood increased, peaking at 4 µg/L. Additionally, it was shown that the odds ratio of urinary urgency incontinence incidence exhibited an upward trend as blood and urine lead levels increased. Notably, with OR reaching its highest point at 7 µg/dL for blood lead levels and 5 µg/L for urinary lead levels. A Cross-sectional study of Yi [24], pp. 2005–2006) examined the correlation between phthalate exposure and urinary tract infection (UI) in 2,821 adults from the United States. The study identified several phthalates that were significantly associated with an increased risk of UI: mono-carboxynonyl phthalate (MCNP), mono-carboxyoctyl phthalate (MCOP), mono-isobutyl phthalate (MiBP), mono-n-butyl phthalate (MBP), and mono-3-carboxypropyl phthalate (MCPP) (*p* value < 0.05). Yang and Liu also [25], pp. 2003–2008) observed that DEHP exposure and OAB in the 2,121 participants from NHANES study. However, all the previous study above only examined some harmful chemical and metal pollutants. There are few studies investigating the effects of common environmental pollutants such as daily fuel on the common urinary incontinence symptoms, which merits further investigation.

In this nationwide investigation, nearly half of (45.98%) participants relied on polluted household fuels to meet their domestic needs in the India. At the same time, as one of the most common diseases of urinary system, urinary incontinence symptoms seriously affect the middle-aged and elderly people life quality in India, especially in elderly females compared to males (UI:55.77% vs. 44.23%; SUI:68.98% vs. 31.02%). In our study, both male and female diagnosed UI or SUI symptoms were substantially correlated with home air pollution. Furthermore, these correlations maintained their statistical significance even after accounting for several possible covariates. Moreover, several significant interaction *P*-value were observed including age, educational level, tobacco, alcohol consumption and physical activity.

Although this study confirmed an association between household air pollution and the likelihood of developing UI/SUI symptoms, the mechanism underlying this relationship remains unclear potentially stemming from oxidative stress (OS) and metabolic disturbances. First, solid fuel accelerates the development of depression and cognitive decline by elevating oxidative stress (OS) in the human body through a high concentration of PM and chemicals. Animal studies have demonstrated that exposure to PM2.5 induces oxidative stress via activation of the Nrf2/NF-κB pathway, leading to an elevation in inflammatory cytokine levels [26]. According to a research on female solid fuel users, there were 32% more leukocytes in the blood, and neutrophils, lymphocytes, eosinophils, and alveolar macrophages all produced more reactive oxygen species (ROS) than clean fuel users [10, 12]. Systemic inflammation and oxygen free radicals can cause oxidative damage to urethra and bladder cells’ membranes, resulting in further urinary incontinence. And inflammation substance and oxygen free radicals can also cause damage to nerve cells, resulting in abnormal nerve conduction function, which may interfere with the signaling of urine control. Another potential association between household air pollution and urinary incontinence (UI)/stress urinary incontinence (SUI) may be attributed to metabolic disturbances. The interaction between PM (particulate matter) and chemical substances has been associated with metabolic disorders, influencing hormonal secretion, autonomic nervous system equilibrium, and vasopressin secretion [8, 11, 13]. The aforementioned pathways play a pivotal role in modulating the regulation of smooth muscle tone, a critical factor influencing the relaxation and contraction dynamics of the detrusor and bladder musculature. Consequently, these pathways are likely associated with the modulation of urinary function. Also, we acknowledging that there may be some potential other pathways that may contribute to the outcomes of household air pollution and UI/SUI symptoms.

Rural India faces significant challenges in accessing clean fuel due to economic constraints, with approximately 40% of Indian households still reliant on unclean biomass fuel for domestic cooking purposes [27]. This underscores the critical role of India’s policy interventions in addressing indoor air pollution. Initiatives such as the National Programme on Improved Chulhas (NPIC) and the PMUY must receive robust support and development from the Indian government. Additionally, there is a pressing need for comprehensive studies to assess and potentially reconsider the pricing of clean fuels, along with subsidies targeted towards economically disadvantaged populations. Policy efforts should also extend to raising awareness about the detrimental effects associated with the use of unclean biomass energy sources and the resulting indoor air pollution.

This research, conducted as part of a nationwide investigation, possesses several limitations. Firstly, the diagnosis of the majority of UI/SUI cases primarily depended on self-report rather than employing more objective examinations, such as urodynamics, which may potentially compromise the accuracy of the diagnoses. Secondly, given the cross-sectional nature of the study, it is plausible that the precise determination of a causal association between household air pollution and symptoms of urinary incontinence (UI)/stress urinary incontinence (SUI) may not precisely determined; However, upcoming prospective and intervention research holds the potential to offer a more thorough elucidation. Additionally, it is noteworthy that certain covariables might be vulnerable to recollection bias.

## Conclusions

Our research found that urinary incontinence/stress urinary incontinence symptoms were significantly linked to the household air pollution among middle-aged and older Indian adults. Moreover, our research has revealed that individuals in the middle-aged and older demographic who utilize solid fuels exhibit a heightened propensity for the manifestation of symptoms associated with urinary incontinence and stress urinary incontinence, in comparison to those employing clean fuels. Given the swift progression of population aging, it is imperative for elderly individuals utilizing solid fuels to expeditiously transition to cleaner energy sources, which is crucial to guarantee their quality of life and physical fitness. Moreover, it is essential to conduct additional prospective studies to clarify the aforementioned correlation and investigate potential underlying mechanisms and evaluate the effectiveness of interventions to mitigate the impact of household air pollution on urinary health.

## Data Availability

Publicly available datasets (Longitudinal Ageing Study in India, Wave 1) were analyzed in this study, which can be found at: https://www.iipsindia.ac.in/content/data-request or https://g2aging.org/app/lasi/download.
